# Direct optic nerve sheath (DONS) application of Schwann cells prolongs retinal ganglion cell survival *in vivo*

**DOI:** 10.1038/cddis.2014.399

**Published:** 2014-10-16

**Authors:** L Guo, B Davis, S Nizari, E M Normando, H Shi, J Galvao, L Turner, J Shi, M Clements, S Parrinello, M F Cordeiro

**Affiliations:** 1 Glaucoma and Retinal Neurodegeneration Research, Visual Neuroscience, UCL Institute of Ophthalmology, London, UK; 2 Western Eye Hospital, Imperial College Healthcare Trust, London, UK; 3 Cell Biology, UCL Institute of Ophthalmology, London, UK; 4 Tongji Hospital, Tongji Medical School, Huazhong University of Science and Technology, Wuhan, China; 5 China-Japan Union Hospital, Jilin University, Changchun, China; 6 Cell Interactions and Cancer, Clinical Sciences Centre, Hammersmith Hospital, Imperial College, London, UK

## Abstract

Cell-based therapies are increasingly recognized as a potential strategy to treat retinal neurodegenerative disease. Their administration, however, is normally indirect and complex, often with an inability to assess in real time their effects on cell death and their migration/integration into the host retina. In the present study, using a partial optic nerve transection (pONT) rat model, we describe a new method of Schwann cell (SC) delivery (direct application to injured optic nerve sheath, SC/DONS), which was compared with intravitreal SC delivery (SC/IVT). Both SC/DONS and SC/IVT were able to be assessed *in vivo* using imaging to visualize retinal ganglion cell (RGC) apoptosis and SC retinal integration. RGC death in the pONT model was best fitted to the one-phase exponential decay model. Although both SC/DONS and SC/IVT altered the temporal course of RGC degeneration in pONT, SC/DONS resulted in delayed but long-lasting effects on RGC protection, compared with SC/IVT treatment. In addition, their effects on primary and secondary degeneration, and axonal regeneration, were also investigated, by histology, whole retinal counting, and modelling of RGC loss. SC/DONS was found to significantly reduce RGC apoptosis *in vivo* and significantly increase RGC survival by targeting secondary rather than primary degeneration. Both SC/DONS and SC/IVT were found to promote RGC axonal regrowth after optic nerve injury, with evidence of GAP-43 expression in RGC somas and axons. SC/DONS may have the potential in the treatment of optic neuropathies, such as glaucoma. We show that SC transplantation can be monitored in real time and that the protective effects of SCs are associated with targeting secondary degeneration, with implications for translating cell-based therapies to the clinic.

In the central (CNS) and peripheral (PNS) nervous systems, injury from initial lesions can lead to widespread damage to neurons beyond the primary injury site; a phenomenon known as ‘secondary degeneration'. Studies in spinal cord injury have revealed secondary rather than primary degeneration to be the major contributor to neuronal death and functional impairment, and it is increasingly recognized as a therapeutic target.^1,2^ Secondary degeneration also occurs in optic neuropathies, including glaucoma, ischaemic optic neuropathy, and Leber's hereditary optic neuropathy.^3, 4, 5^ Retinal neuronal loss in these conditions is reported to occur long after the initial insult,^6^ implying that secondary mechanisms may have an important role in optic neuropathic damage and that targeting of secondary neuronal loss may represent a novel therapeutic strategy.

Partial optic nerve transection (pONT) represents a reliable and reproducible model for studying secondary degeneration, in which a primary lesion is only made to dorsal axons and leaves those in ventral optic nerve (ON) intact but vulnerable to secondary degeneration.^4,7^ Secondary degeneration is thought to be initiated by a cascade of reactive metabolic events, including glutamate excitotoxicity, Ca^2+^ overload, excess free radical formation, oxidative stress, mitochondrial dysfunction, and increased proteoglycan expression, leading to cell death.^7, 8, 9, 10, 11, 12, 13, 14^ Activated astrocytes are reported to be a major contributor to spreading and acceleration of secondary degeneration.^8,9^

As in most CNS pathways, the mature ON possesses only a limited ability to repair itself after injury, resulting in permanent vision loss due to the death of retinal ganglion cells (RGCs), the retinal output neurons that transmit visual information to the brain.^15^ Compared with the CNS, the PNS has a remarkable ability to regrow after injury, a process in which Schwann cells (SCs) are thought to have a key role.^16,17^

SCs are the principal glia of the PNS and support normal neuronal function.^18,19^ Upon axonal injury, SCs are reported to shed their myelin sheaths and de-differentiate into progenitor stem cells, which are capable of replacing damaged tissue and providing a permissive environment for neuronal survival and axonal regrowth.^18,19^ SCs are believed to achieve this through releasing neurotrophic factors and producing cell adhesion molecules and extracellular matrix components.^20^ The neuroprotective and regenerative mechanisms between SCs and neurons are thought to operate on a local basis via adhesion molecules, allowing contact-mediated signalling between cells,^16,17,20,21^ and extracellular free ligands, facilitating specific binding to the receptors in the target neurons.^16,17,20^ However, a novel regulatory mechanism has emerged, representing a more efficient and advanced communication machinery, that is, vesicular transfer between SCs and axons.^16^ We have recently demonstrated that the highly efficient response of SCs to PN injury is triggered by Ephrin-B/EphB2 signalling in fibroblasts, which guide SC sorting and migration during nerve repair.^21^

Due to the regenerative ability of SCs in PNS repair, transplantation of SCs to the injured ON has been previously attempted.^22, 23, 24, 25, 26, 27, 28^ To date, however, the protective effects of SCs on retinal neurons have been only assessed after either intravitreal administration or suturing artificial SC grafts onto transected ON, using postmortem histological observations, with incomplete delineation of the mechanisms involved.^22, 23, 24, 25, 26, 27, 28^

Here we use a pONT model to investigate a new method of SC delivery (direct application to injured ON sheath, SC/DONS), using *in vivo* imaging and histological techniques, and compare its effects on RGC apoptosis and loss to intravitreal SC delivery (SC/IVT). Furthermore, we analyse whether these actions target primary or secondary degeneration, to determine their potential in the treatment of optic neuropathy.

## Results

### SC/DONS reduces *in vivo* RGC apoptosis

**Figure 1:**
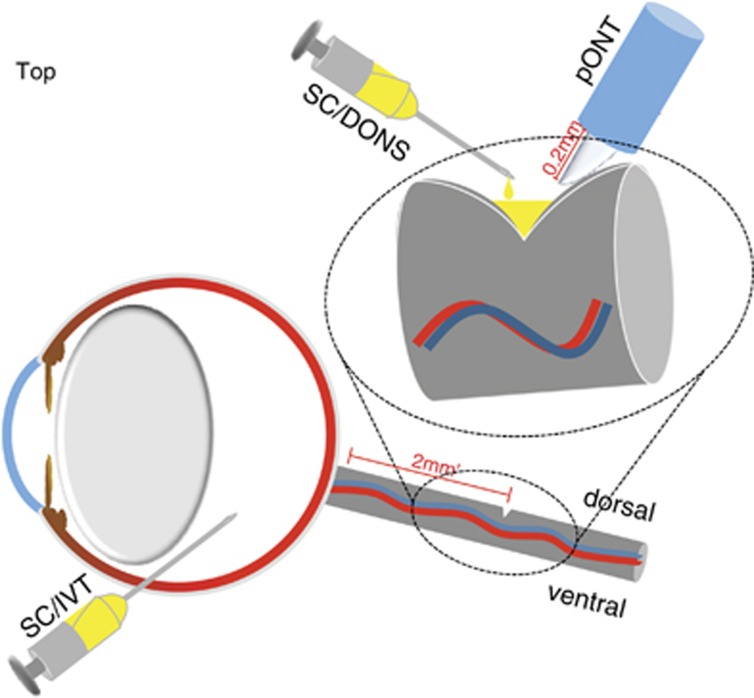
Diagram of administrations of SCs in pONT. Schematic diagram shows SC/DONS and SC/IVT transplantation of SCs in a rat pONT model. SCs were administered directly to the ON sheath in the SC/DONS application after pONT (0.2 mm cut was made in the dorsal optic nerve), whereas with SC/IVT SCs were injected into the vitreous 3 days before pONT

**Figure 2:**
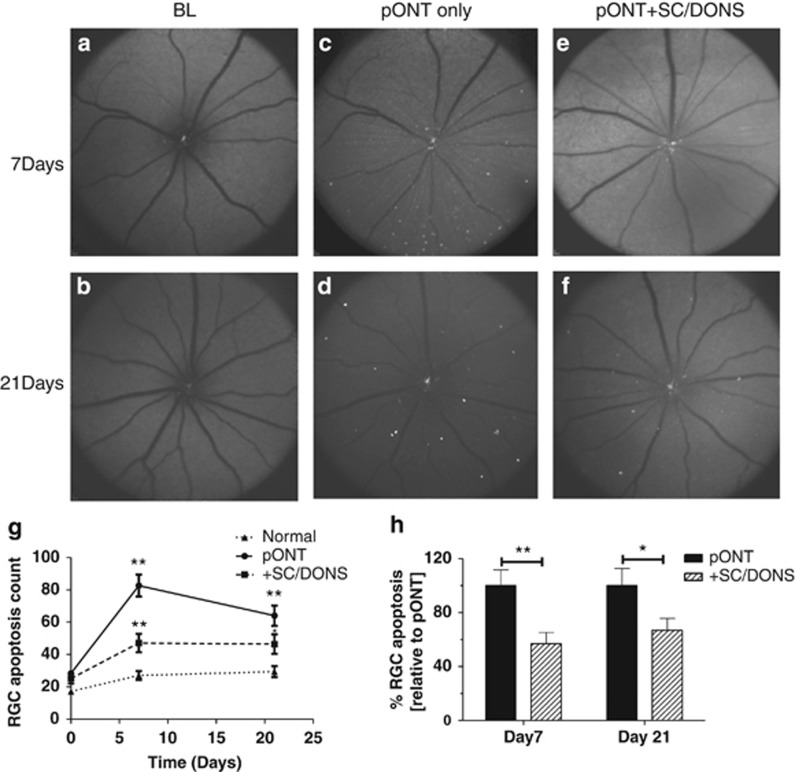
SC/DONS application of SCs reduces *in vivo* RGC apoptosis. RGC apoptosis was labelled by intravitreal injection of fluorescently-labelled annexin V and imaged using a cSLO. Representative DARC images show RGC apoptosis (white spots) at baseline (**a** and **b**), pONT only (**c** and **d**), and SC/DONS-treated pONT (pONT+SC/DONS, **e** and **f**) at 7 (**a**, **c**, **e**), and 21 (**b**, **d**, **f**) days following treatment. pONT induced a significant increase of RGC apoptosis at 7 and 21 days, compared with normal control (**g**) and SC/DONS treatment significantly reduced RGC apoptosis at both 7 and 21 days compared with untreated group (pONT) (**g** and **h**). Data were presented as means±S.E. *P<0.05, **P<0.01

To minimize invasive procedures, SC/DONS was only made once during the surgery of pONT. SCs (2 × 10^8^cells/ml) were mixed with Matrigel in a 1 : 1 ratio immediately before application, and 5 *μ*l of the mixture was administered onto the injured ON at the end of pONT surgery (Figure 1). Having previously shown that ON injury can be evaluated *in vivo* using DARC (Detection of Apoptotic Retinal Cells),^29^ we first assessed the effects of SC/DONS with the same method in pONT. Compared with baseline (Figures 2a and b), untreated pONT induced a significant increase in RGC apoptosis at both 7 (Figure 2c, maximal level) and 21 (Figure 2d) days (Figure 2g, *P*<0.01), which was similar to our previous study.^29^ SC/DONS significantly reduced RGC apoptosis at both 7 (Figures 2e, g–h, *P*<0.01) and 21 (Figures 2f, g–h, *P*<0.05) days compared with untreated pONT. In this experiment, the percentage of RGC apoptosis relative to SC/IVT could not be evaluated due to an overlap of the fluorescence spectra of green fluorescent protein (GFP)-SCs and apoptotic RGCs labelled with annexinV-488. In addition, this study only assessed SC effects following one application during pONT surgery, in order to minimize the number of invasive and anaesthetic procedures. However, to prolong therapeutic efficacy and to mimic the clinical situation, we recognize the need to assess different time points of DONS application. This is to be the subject of future work.

### Establishment and validation of a novel algorithm for RGC count

**Figure 3:**
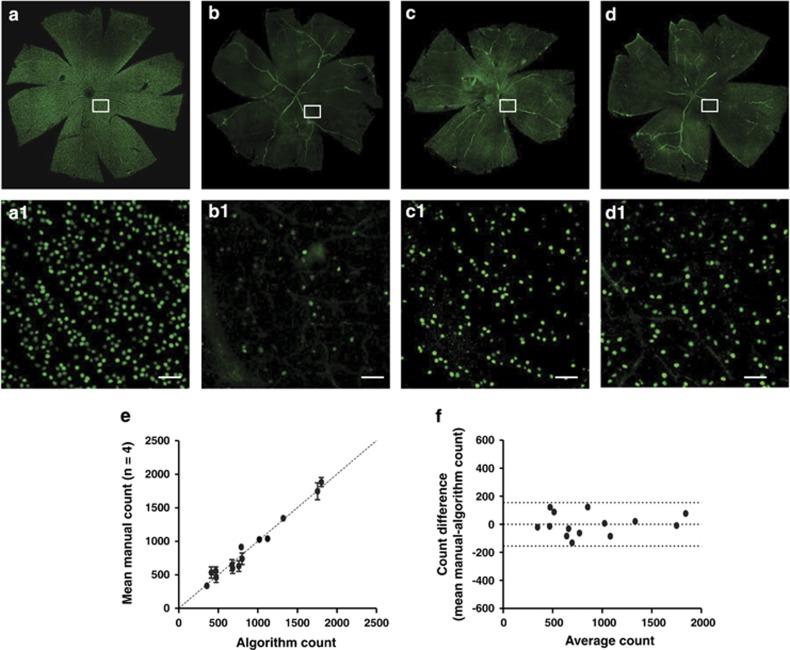
SC effects on RGC survival after pONT can be evaluated using an algorithm on whole retinal mounts. RGCs in the whole retina were labelled with Brn-3a antibody and imaged by confocal fluorescence microscopy. (**a**) Normal retina, (**b**) pONT retina, (**c**) pONT+SC/DONS retina, (**d**) pONT+SC/IVT retina, and (**a**1, **b**1, **c**1 and **d**1) represent corresponding areas of high magnification images at 21 days following pONT. A substantial loss of RGCs was noted in the pONT only retina (**b** and **b**1), compared with the normal retina (**a** and **a**1), and SC/DONS treatment (**c** and **c**1) clearly reduced RGC loss. This increase in RGC survival was also seen in SC/IVT-treated eyes (**d** and **d**1). The number of RGCs in retinal whole mounts was analysed using our newly established algorithm in ImageJ. To validate the algorithm, RGCs in 0.92 mm^2^ retinal sections were manually counted by four trained individuals. (**e**) Correlation of algorithm output to average manual counting fitted a straight line with a correction factor based on a previous study^30^ (Pearson's correlation coefficient=0.9860, *P*<0.0001, *R*^2^=0.9722). (**f**) Bland–Altman plot shows good agreement between algorithm and manual counts after correction. Scale bars: 50 *μ*m

Brn-3a-positive-stained cells were visualized in retinal whole mounts using confocal microscopy (Figures 3a–d). Automated counts using the algorithm were compared with that obtained manually with four trained observers on normal whole retina, in 0.92 mm^2^ retinal areas (*n*=14). A correction factor was applied to mitigate proportional bias in the algorithm,^30^ from which a highly significant correlation between the automated and manual RGC counts was obtained (Figures 3e and f and Supplementary Figures S1a–c, Pearson's correlation coefficient=0.9860, *P*<0.0001, *R*^2^=0.9722). With the algorithm counting, the total number of RGCs in normal retina was 80 818±4919, and RGC density was 2152±526 (cells/mm^2^), which are comparable to the estimates from previous studies.^30,31^

### SC/DONS enhances RGC survival relative to SC/IVT

**Figure 4:**
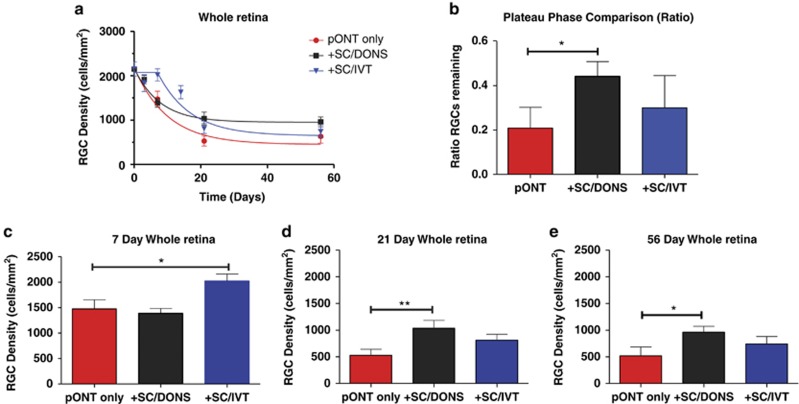
SC/DONS application promotes RGC survival longer than SC/IVT. (**a**) RGC loss in pONT only (red line) and pONT+SC/DONS treatment (black line) fitted to a one-phase exponential decay model with plateau (Equation 1), while pONT+SC/IVT treatment (blue line) fitted to a model of plateau followed by a one-phase decay (Equation 2). (**b**) SC/DONS (but not SC/IVT) treatment significantly increased the plateau of RGC density, compared with the pONT only group, suggesting a significant neuroprotective effect. (**c**–**e**) SC/IVT treatment significantly increased RGC survival at 7 days (**c**) while SC/DONS treatment increased RGC survival at 21 (**d**) and 56 (**e**) days, compared with pONT only. Data were presented as means±S.E. *P<0.05; **P<0.01

Using the validated algorithm, we next assessed the effects of SC transplantation on RGC survival following pONT. RGC density counts were used to create longitudinal profiles of RGC loss following pONT, which was fitted to a one-phase exponential decay model with plateau (Equation 1) (Figure 4a, red line).


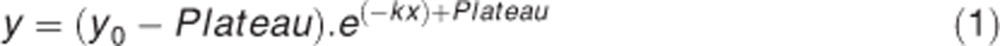


SC/DONS significantly increased the plateau phase (Figure 4a, black line) of the ratio of remaining RGC (Figures 4b, *P*<0.05) and RGC density from 21 (Figure 4d, *P*<0.01) to 56 (Figure 4e, *P*<0.05) days compared with pONT only group. This profile was next compared with that seen in SC/IVT, which in comparison best fitted to a model of plateau followed by a one-phase decay (Equation 2) (Figure 4a, blue line). A significant protective effect was observed only at 7 days with SC/IVT (Figure 4c, *P*<0.05), compared with pONT only.


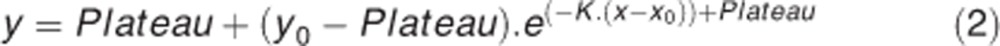


To ensure that protective effects on RGCs were a result of SC-mediated neuroprotection rather than Matrigel (for DONS) or culture medium (for IVT) derived growth factors; the effects of vehicle on RGC survival following pONT were assessed parallel to the treatments of SC/DONS and SC/IVT. Both Matrigel and culture medium treatments were shown to have no significant protective effect, compared with pONT only animals (Supplementary Figures S2a and b, *P*>0.05). By contrast, both SC/DONS and SC/IVT resulted in a significant increase on RGC density compared with pONT only (Supplementary Figures S2a and b, *P*<0.01, *P*<0.05).

### SC/DONS predominantly target RGC secondary degeneration with prolonged effects compared with SC/IVT

**Figure 5:**
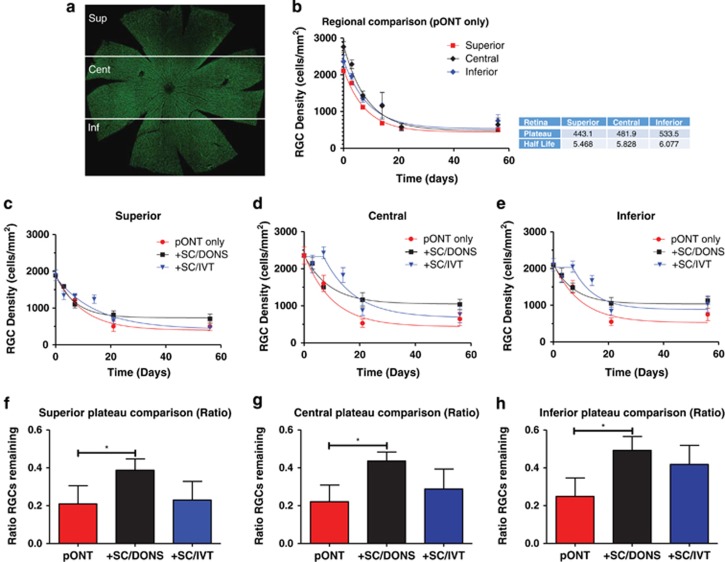
SC/DONS application promotes RGC survival by targeting secondary degeneration. (**a**) Confocal retinal whole-mount, stained with Brn-3a, was divided into the superior, central, and inferior regions, and RGC density calculated for each region. (**b**) Profiles of pONT-induced RGC loss in the superior, central, and inferior retina fitted to one-phase exponential decay model (Equation 1) and showed no significant difference among the three regions in half-lives and plateau, indicating that secondary degeneration occurred almost simultaneously with primary damage. (**c**–**e**) The effects of SC transplantation on RGC density in the superior (**c**), central (**d**), and inferior (**e**) retina reveal that SC/DONS showed more protection in the late stages, with a significant increase of plateau in the superior (**f**), central (**g**), and inferior (**h**) retina compared with the no treatment group. In comparison, SC/IVT showed an early delay in RGC loss followed by a rapid decay in the central and inferior retina (**d** and **e**). Data were presented as means±S.E. *P<0.05

Having established that SCs promoted RGC survival after pONT, we next investigated whether the protective effects were associated with inhibiting primary or secondary degeneration. pONT injures the dorsal ON fibres, inducing RGC primary degeneration in the superior retina, while the RGCs in the central and inferior retina survive the initial insult but are vulnerable to secondary degeneration.^4,7^ To separate primary and secondary events and avoid bias in RGC counts, retinal whole mounts were divided into the superior, central, and inferior regions, and RGC densities were calculated for each region (Figure 5a). In pONT only eyes, the longitudinal profiles of RGC loss in all three regions best fit to a one-phase exponential decay model (Equation 1), with a slight but not significant increase in half-lives of 5.5, 5.8 and 6.1 days and plateaus of 443.1, 481.9 and 533.5 in the superior, central, and inferior retina, respectively (*P*>0.05, Figure 5b).

RGC profiles (RGC density and the ratio of RGCs remaining to normal controls) in SC/DONS fitted to a one-phase exponential decay model in all three regions (Figures 5c–e, black line), demonstrating significantly greater protection in the late stages, with a significant increase in the plateau phase in all three regions (Figures 5f–h, *P*<0.05). In comparison, SC/IVT in the central and inferior regions (Figures 5d and e, blue line) was best fitted to a model of plateau followed by a one-phase decay (Equation 2), in which a delayed loss of RGCs was observed in the early stage followed by rapid RGC loss.

RGC survival was predominant in the central and inferior regions, where RGCs are vulnerable to secondary degeneration, but not in the superior region, where primary degeneration is believed to be predominant (Supplementary Figure S3). SC/DONS resulted in significant protective effects at 21 and 56 days (Supplementary Figures S3b and c, *P*<0.05, *P*<0.01). In comparison, SC/IVT showed an early effect at 7 days until 21 days (Supplementary Figures S3d and e, *P*<0.05). These results suggest that transplanted SCs mainly affect secondary degeneration, and SC/DONS shows a delayed but prolonged protective effect compared with SC/IVT.

### Transplanted SCs integrate into the retina and ON and promote axonal regrowth

**Figure 6:**
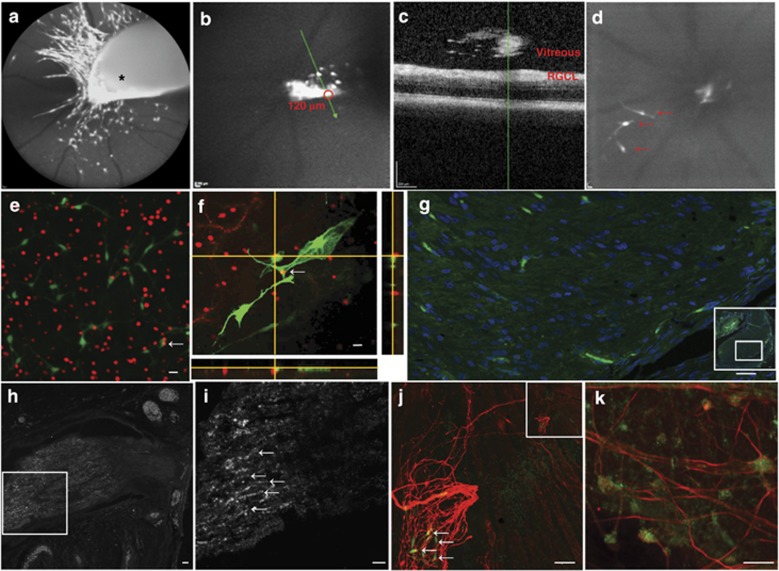
Transplanted SCs can migrate and integrate into the retina and the optic nerve and promote RGC axonal growth. (**a**) *In vivo* cSLO image of GFP-SCs revealed a big cluster of cells within vitreous cavity (*) 3 days following transplantation, with some cells appearing to elongate and migrate towards the retina. (**b** and **c**) Combining *in vivo* cSLO (**b**) with OCT (**c**) technology allows anatomical localization of GFP-SCs with an ‘optical section' defined by the cSLO image (SC cluster outlined with red circle, with position of OCT slice indicated by green line, **b**) and visualized using the OCT (corresponding position shown by green line, **c**). (**d**) *In vivo* cSLO image shows GFP-SCs close to, with an elongated morphology, and integrating within the retina at 21 days (arrows) but at a reduced number. (**e** and **f**) Confocal fluorescence microscopy of whole retinal mounts showing GFP-SCs (green) integrating into the RGC layer (RGCL, Brn-3a labelled, red) at 3 (**e**) and 21 (**f**) days after pONT (yellow, arrows and *z*-stack image of the RGCL). Transplanted cells appeared to become elongated at 21 days, similar to what is seen *in vivo*. (**g**) GFP-SCs (green) integrated and migrated in the injured optic nerve 21 days after SC/DONS transplantation (inset: smaller magnification of the ON). (**h** and **i**) GAP-43-labelled newly growing axons (white) in an injured ON after SC transplantation at low (**h**) and high (**i**) magnification. (**j**) Neurofilament-L-labelled regenerated axons (red) in the retina, showing co-localization (arrows) with transplanted GFP-SCs (green) 14 days following pONT. The inset is a low magnification of the area showing the optic nerve head. (**k**) GAP-43-labelled RGCs (green) with neurofilament-L-labelled axons (red) 21 days following ON injury and SC transplantation. Scale bars: 20 *μ*m

To monitor the fate of SCs after SC/IVT, imaging was performed *in vivo* using the HRA (Heidelberg retina angiograph)+OCT (optical coherence tomography) Spectralis. GFP-SCs were visualized for at least 21 days in the vitreous, with most cells located around the posterior capsule of the lens.^32^ A small proportion of the cells migrated close to or on to the retina as early as 3 days following transplantation (Figure 6a). By using the *in vivo* cSLO (confocal scanning laser ophthalmoscopy) image (Figure 6b) to define an area of interest, it is possible to localize anatomically SC migration in the vitreous, closed to the retina in an OCT-acquired optical section (Figure 6c). GFP-SCs were migrating close to and integrating into the retina at 21 days (arrows) but at a reduced number. These *in vivo* observations were validated by confocal histology (Figures 6e and f) where GFP-SCs are clearly visible in the RGCL. With SC/DONS, SCs were observed to migrate into the injured ON where they integrated within the axonal bundles (Figure 6g). RGC axonal regrowth was identified in the injured ON after SC/DONS (Figures 6h and i) and in the retina (Figure 6j) following SC/IVT, which was consistent with the overexpression of growth-associated protein (GAP)-43 in the RGC soma (Figure 6k). In addition, co-localization of regenerated axons and transplanted SCs was seen (Figure 6j). RGC regeneration was not seen in control animals with pONT only (data not shown).

## Discussion

We have demonstrated a new approach to applying SCs directly to the ON sheath (SC/DONS) and have shown it to significantly reduce RGC apoptosis and prolong RGC survival compared with IVT application following pONT in the rat. Our results also show that transplanted SCs can be monitored *in vivo*, and they are able to integrate into the RGCL and injured ON, promoting RGC axonal regrowth. Finally, we show that the protective effects of SCs are associated with targeting secondary degeneration, with implications for translating cell-based therapies to the clinic.

ON axotomy is often used to model optic neuropathies,^4,5^ which clinically are a leading cause of irreversible blindness due to degeneration and loss of RGCs. Such neuropathies include glaucoma, ischaemic optic neuropathy, and Leber's hereditary optic neuropathy.^3, 4, 5,33, 34, 35^ Protection and regeneration of RGCs and their axons following injury is a clear unmet medical need.

Transplantation of SCs has been previously attempted intravitreally or by grafting onto an axotomized ON, with successful regrowth and enhanced RGC survival histologically.^20,23, 24, 25, 26,36^ However, these models involved transplantation of SCs to a completely transected ON, in which artificial SC grafts were inserted into the stumps of cut ON.^22, 23, 24, 25, 26, 27, 28^ In the present study, we have attempted, for the first time, to directly apply SCs to a partially transected ON sheath in rat. We found that SCs, with the aid of Matrigel, enables not only migration into the lesion site but also penetration and integration into the injured ON. Matrigel, mainly comprising laminin and collagen IV, has been shown to be supportive for SC survival and elongation *in vitro*^37^ and *in vivo*,^38^ possibly due to the presence of growth factors.^39^ However, Matrigel alone was found not to protect against pONT-mediated RGC degeneration, with no significant difference in RGC density observed between pONT only and pONT+Matrigel. Compared with artificial grafts, the SC/DONS appears to be an easier and potentially more practical way of treating ON injury, and the protective effect is more significant than intravitreal administration.

The ability to use real-time imaging methods to monitor effects and efficacy of treatment, as shown in this study, highlights the translatable aspects of the results. Previously, we have only used DARC to assess complete axotomy^29^—but interestingly, in that model, we also showed peak apoptosis to occur at 7 days, suggesting a similar temporal profile and course of RGC death. Similar results were found by Wang *et al.*^40^ recently, using histological analysis in the same pONT model, with peak RGC apoptosis at 1 week which gradually decreased over 12 weeks. One study showed different results, however, which may be attributed to different experimental protocols. First, Levkovitch-Verbin *et al.*^13^ applied a milder insult cutting the ON to a depth of 0.1 mm compared with 0.2 mm in this study and approximately 0.4 mm in the study by Wang *et al.*^40^—we and the others have previously shown that the extent of neurotoxicity is dependent on the magnitude of the insult.^41,42^ Second, rat species and weights were different.^31^ Finally, the method used to identify apoptosis also differed: Hoechst staining in histological cross-sectional samples in the study by Levkovitch-Verbin *et al.*, indirect estimate by Wang *et al.*,^40^ and the *in vivo* demonstration of annexin V-positive cells used in our study.

Manual counting of total RGCs is time consuming, labor intensive, and tedious, with potential vulnerability to bias. Sampling counting of RGCs from selected areas of the retina is therefore chosen by many researchers.^4,7,40,43,44^ However, it is evident that considerable variability exists even if a significant fraction of the retinal area (quadrants or hemi-retinas) are being sampled.^30^ In the present study, we have established a simple algorithm to automatically count RGCs in the entire retina and have shown that the algorithm output is comparable to manual counting. The total number of RGCs in normal Dark Agouti (DA) rats obtained from the algorithm count (80 431 cells) is comparable to other published automated counts of 81 486 in SD rats, 87 229 in PVG rats^31^ and 97 609 in Wistar rats,^30^ and is also comparable to the estimated total number of RGCs by sampling manual counts of 87 809^4^ and 74 104^45^ in Wistar rats and 77 400 in SD rats.^46^ The variations of RGC counts in different studies can be caused by both animal strains^31^ and methodologies, such as RGC markers and sampling method employed.^30^

A general phenomenon shared by many forms of neurodegeneration is a continuous steady or exponential decline in neuronal number over time.^47,48^ This one-hit model of cell death has been proposed in inherited and acquired neuronal degenerative diseases, with implications for therapeutic strategies.^47,48^ Previous studies have attempted to apply an exponential decay model to the RGC loss occurring in ON crush and complete transection in Thy-1 CFP mice.^49,50^ Here we report that RGC death in the pONT model is best fitted to the one-phase exponential decay model. Our results reveal that although both DONS and IVT transplantation routes of SCs alter the temporal course of RGC degeneration in pONT, they act in different ways: SC/DONS results in a significant increase in the duration of the plateau phase (increasing RGC survival) while SC/IVT delays the early phase of degeneration followed by a rapid decay. The mechanisms behind the difference are unknown, but the earlier effects of SC/IVT could be due to them producing neurotrophic factors locally that rapidly affect surrounding RGCs.^20,27,28,51^ Furthermore, the short-lived duration (<56 days) of effect may be due to the rapid decline in the SC number in the vitreous. The delayed but long-lasting effects of SC/DONS could be attributed to their interaction and signalling with injured RGC axons, as direct contact of ON axons with living SCs has been shown to have an important role in RGC survival and regeneration.^52,53^

Partial ONT provides a good model for studying secondary degeneration,^4,7,8,13,54, 55, 56^ which is defined as occurring after an initial insult, consisting of widespread neuronal loss and severe functional impairment far in excess of the original injury.^1,55^Limiting secondary degeneration following a primary insult is practically the only option in chronic disease such as glaucoma, where initially the patient is asymptomatic.^57^ We demonstrate that SCs improve RGC survival predominantly through inhibition of secondary rather than primary degeneration, as evidenced by the significant preservation of RGC density in the central and inferior but not the superior retinal segment. To the best of our knowledge, this is the first time that SC's neruoprotective role in targeting secondary degeneration has been demonstrated. This suggests that the therapeutic potential of SC transplantation may be more practical for translating to the patient, because clinical management of chronic ON damage is aimed at reducing secondary damage after the onset of disease.^57,58^

It appears in our study, that in untreated pONT, where RGC degeneration in all three regions is best fitted to a one-phase exponential decay model, the kinetics of RGC decline (i.e., the plateau and half-life) are extremely similar among the three regions. This suggests that secondary degeneration in this model occurs almost simultaneously with the primary injury. This is in agreement with several recent studies.^9,13,14^ Indeed, secondary degeneration has been demonstrated to be an early event after pONT, with evidence of rapid spread of oxidative stress beyond the injury site within 5 min, and increased production of reactive oxygen/nitrogen species and mitochondrial changes reported by 24 h.^9, 10, 11^ Injury-induced activation of astrocytes is attributed to the early event, properly via spread of oxidative stress and amplification of calcium signals throughout the ON vulnerable to secondary degeneration.^8,9,59^ In support, Ca^2+^ channel blockers have been shown to limit secondary degeneration, resulting in reduced RGC death and preserved visual function.^7,12,59^ In addition, therapeutic modulation of mitochondrial function by red/near-infrared irradiation therapy shows reduced oxidative stress and mitochondrial dysfunction in ON vulnerable to secondary degeneration.^11,60^

*In vivo* monitoring of stem cells after transplantation is essential for a better understanding of their migration/integration into the host tissue. Using clinically available instruments, we demonstrate that this is achievable using *in vivo* retinal imaging. More importantly, we have demonstrated the use of OCT imaging in tracking the fate of clusters of transplanted cells. The findings of the low efficiency of retinal graft integration could be associated with specific barriers, such as reactive gliosis, local inflammatory cells, and extracellular matrix components, and targeting these barriers has the potential to dramatically improve transplanted cell integration.^61, 62, 63, 64^

In accordance with previous studies,^22, 23, 24, 25, 26, 27, 28^ we identify SC-mediated RGC regeneration in both the injured ON and retina. Furthermore, the proximity of transplanted cells and re-growing axons observed in this study supports the notion that physical contact of living SCs with injured RGCs promotes axonal regeneration.^53^ In the PNS, effective communications between injured nerve and local SCs are essential for nerve regeneration, which relies on various regulatory mechanisms, including neurotrophic factors, adhesion molecules, extracellular free ligands, and transfer of vesicles from SCs to axons.^16,20^ The mechanisms behind SC-mediated RGC axonal regeneration observed in this study remain unknown, but it is likely that SCs communicate with injured ON in a similar way to that in the PNS.^16^ This is possibly the most viable explanation as to why SC/DONS was more effective than SC/IVT at preserving RGCs for a prolonged period in the pONT model.

## Materials and Methods

### Animals

Adult male Dark Agouti rats weighing 150–200 g were treated with procedures approved by the UK Home Office and in compliance with the ARVO Statement for the Use of Animals in Ophthalmic and Vision Research. All animals (*n*=105) were maintained in a 12-h light/12-h dark cycle with a room illuminance of 140–260 lux during the bright portion of the cycle. Animals were provided standard food and water *ad libitum*.

### pONT

All animals had pONT performed in the left eye, using a previously described technique.^4^ Under general anaesthesia (GA), an incision was made in the superior conjunctiva, and the ON sheath was exposed. A longitudinal slit was next formed in the dura mater to expose the ON, and a 0.2-mm cut was made in the dorsal ON, 2 mm behind the eye using an ophthalmic scalpel with a steel cutting guard (Figure 1). Damage to major ophthalmic blood vessels was avoided and verified at the end by ophthalmoscopy.

### SC culture

A GFP-transfected SC line,^21,65^ originally derived from rat sciatic nerve, was cultured in DMEM 1.0 g/l glucose medium supplemented with 3% stripped and heat-inactivated foetal calf serum, 4 mM l-glutamine, 1 *μ*M forskolin, 2 *μ*g/ml gentamycin, 0.1 mg/ml Kanamycin, and 0.5 ng/ml Heregulin.^21,65^ Cells were cultured at 37 °C in a humidified incubator with 5% CO_2_ until 80–90% confluency before passaged using 0.25% Trypsin-EDTA.

### SC transplantation

Two modes of SC application were assessed (Figure 1): for SC/DONS application, SCs (2 × 10^8^cells/ml) were mixed with Matrigel (BD Biosciences, Bedford, UK) in a 1 : 1 ratio, and 5 *μ*l of the mixture was applied to the partially transected ON at the end of pONT surgery. For SC/IVT, 5 *μ*l of SCs at 2.5 × 10^8^ cells/ml were injected intraocularly 3 days before pONT surgery. The number of cells administered was calculated based on previous studies elsewhere.^26, 27, 28^ In the control groups, pONT was carried out either without any treatment or using vehicle control. Animals were killed at 3, 7, 14, 21, and 56 days following pONT; 4–10 animals were included in each treatment group at each time point.

### *In vivo* imaging of RGC apoptosis with DARC

Animals with SC/DONS treatment and untreated controls were imaged at baseline for RGC apoptosis before pONT and at 3, 7, 14, 21, and 56 days after pONT using DARC.^29,66, 67, 68, 69^ Briefly, animals under GA were intravitreally injected with fluorescently labelled annexin as previously described,^29,66, 67, 68, 69^ 2 hours before imaging with a cSLO (HRA Spectralis, Heidelberg Engineering, Heidelberg, Germany). The retinal images were then collected, and the number of apoptotic RGCs were manually counted by four trained individuals masked to the treatment groups.^66^

### *In vivo* imaging of SC survival and migration

To monitor SC (GFP-labelled) fate after intravitreal transplantation, animals with SC/IVT transplantation were imaged *in vivo* using a customized HRA+OCT Spectralis^70^ to spatially track GFP-SCs for survival and migration with an argon laser wavelength of 488 nm.

### Immunohistochemistry and confocal microscopy

After animals were killed, both eyes were enucleated and fixed in fresh 4% paraformaldehyde at 4 °C overnight. Retinal whole mounts and ONs were then dissected. To assess RGC survival, retinal whole mounts were stained with an anti-mouse Brn-3a mAb (1 : 500, Merk Millipore, Darmstadt, Germany) and examined under confocal microscopy (LSM 710, Carl Zeiss MicroImaging GmbH, Jena, Germany), to generate a single plane maximum projection of the RGC layer in each retina. Additionally, some retinas were stained with a GAP-43 anti-mouse mAb (1 : 200, Santa Cruz Biotechnology, Inc., Heidelberg, Germany) and neurofilament-L anti-rabbit mAb (1 : 200, New England Biolabs, Hitchin, UK) to assess newly growing RGC axons. The paraffin-embedded ON was sectioned longitudinally and immunostained with GAP-43 mAb (1 : 200) to identify regenerated axons. Confocal microscopy was also used to visualize transplanted GFP-SC migration/integration.

### Automated quantification of Brn-3a labelled RGCs in retinal whole mounts

A newly established algorithm using ImageJ (National Institute of Health, Bethesda, MD, USA) was used to objectively assess RGC counts in retinal whole mounts. Briefly, a high-pass filter was applied to the 8-bit Brn-3a (Green) channel to remove background followed by application of a 130 intensity threshold. The ImageJ watershed algorithm was then used to separate touching particles. Only the particles within 7–21 *μ*m size range were counted based on RGC sizes previously reported.^30^ To exclude blood vessels that also stained with Brn-3a, circularity of each particle was determined using Equation 3 where *A* is the particle area and *P* is the perimeter, and particles with circularity <0.7 were excluded from the final RGC count:


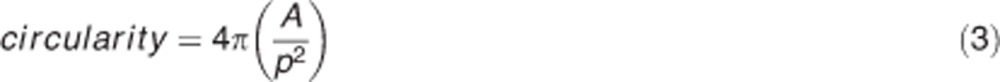


To ensure the validity of the algorithm, counts obtained from the algorithm were compared with manual counts in 0.92 mm^2^ retinal sections by four trained individuals masked to the algorithm results. The algorithm counting could be further improved by introduction of a correction factor (Equation 4), as previously described,^30^ where *D*_*A*_ is actual RGC density (RGC/mm^2^), *D*_*R*_ is the reported RGC density, and *A* is the total retinal area (mm^2^) (Equation 4).


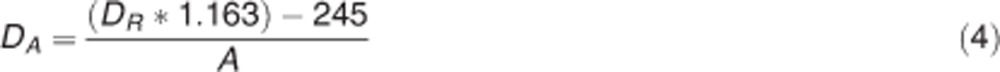


To calculate RGC density (cells/mm^2^), the area of each retina was measured using ImageJ, and the algorithm counts were divided by retinal area (mm^2^). The ratio of the average density of RGCs to untreated controls (0–1) was also used for assessing SC treatment. To assess SC effects on primary and secondary degeneration, retinal images were divided into three equal parts of the superior, central, and inferior retina before RGC density was calculated for each region.

### Statistical analysis

All data were analysed with the Student's *t*-test or one-way ANOVA as appropriate using GraphPad Prism 5 (GraphPad Software, Inc., La Jolla, CA, USA). The longitudinal profiles of RGC degeneration following pONT and SC treatment were fitted with either a one-phase exponential decay model with plateau (SC/DONS, Equation 1 in Results section) or a model of plateau followed by a one-phase decay (SC/IVT, Equation 2 in Results section). Data were presented as means±S.E., and *P*<0.05 was considered to be significant.

## Abbreviations

CNS: central nervous system
PNS: peripheral nervous system
pONT: partial optic nerve transection
ON: optic nerve
RGC: retinal ganglion cell
SC: Schwann cell
DONS: direct optic nerve sheath
IVT: intravitreal treatment
GFP: green fluorescent protein
HRA: Heidelberg retina angiograph
cSLO: confocal scanning laser ophthalmoscopy
OCT: optical coherence tomography
GAP: growth-associated protein
GA: general anaesthesia
