# Supplementary figures and images for: Direct optic nerve sheath (DONS) application of Schwann cells prolongs retinal ganglion cell survival in vivo
Source: Cell Death Dis. 2014 Oct 16;5(10):e1460–. doi: 10.1038/cddis.2014.399 (PMC4237238; doi:10.1038/cddis.2014.399)

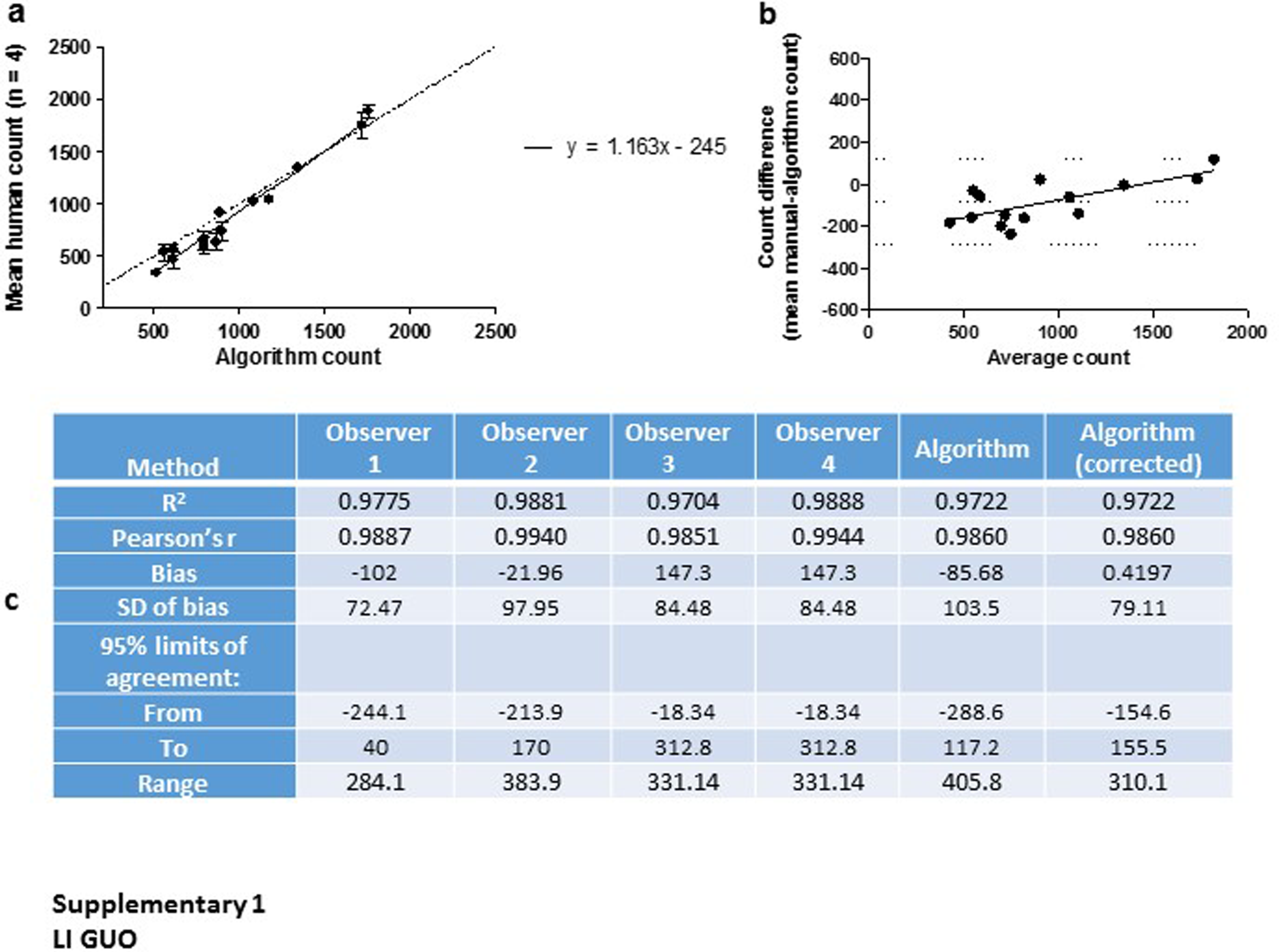

Supplement: Supplementary S1 [file cddis2014399x1.tif]

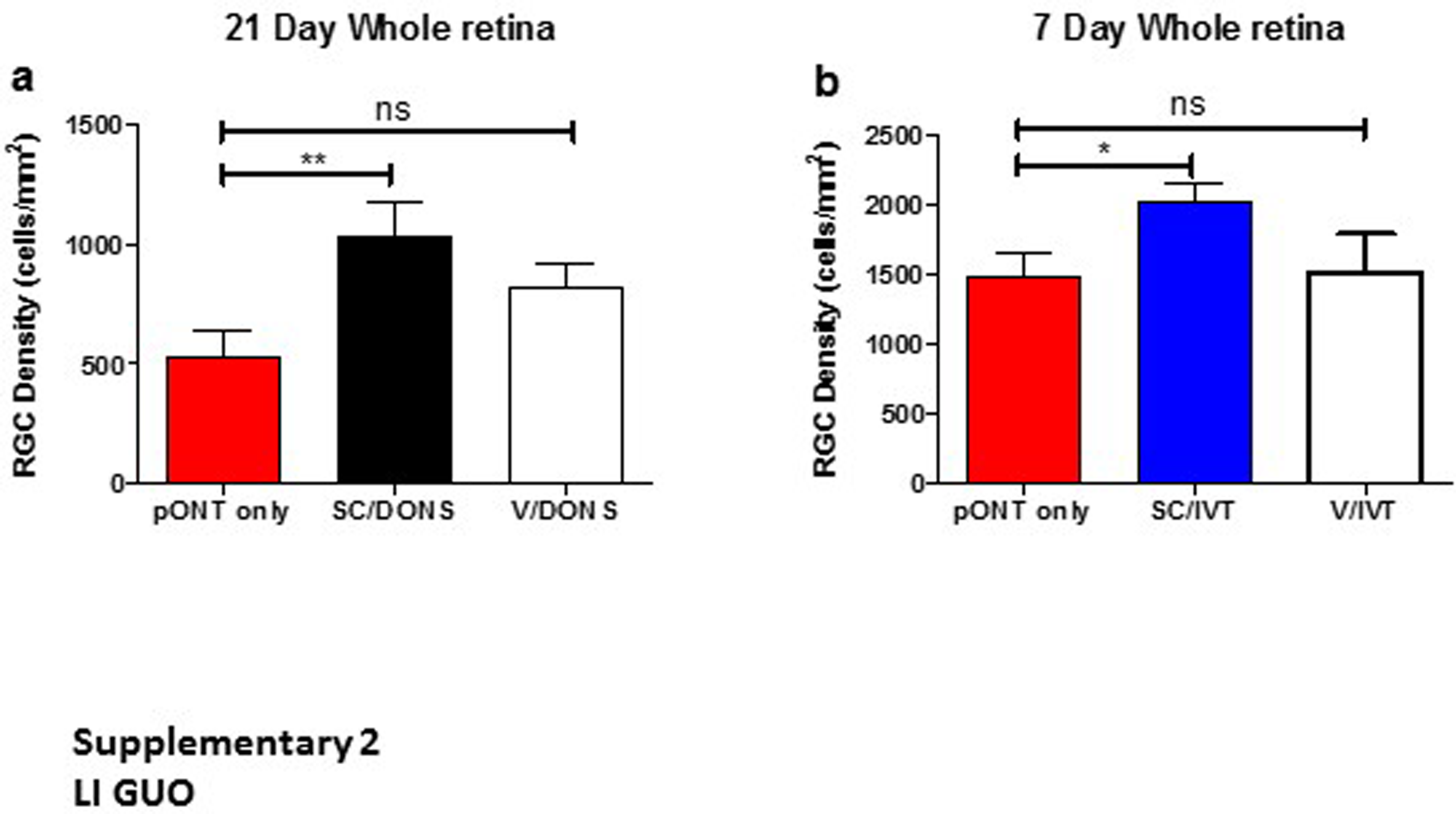

Supplement: Supplementary S2 [file cddis2014399x2.tif]

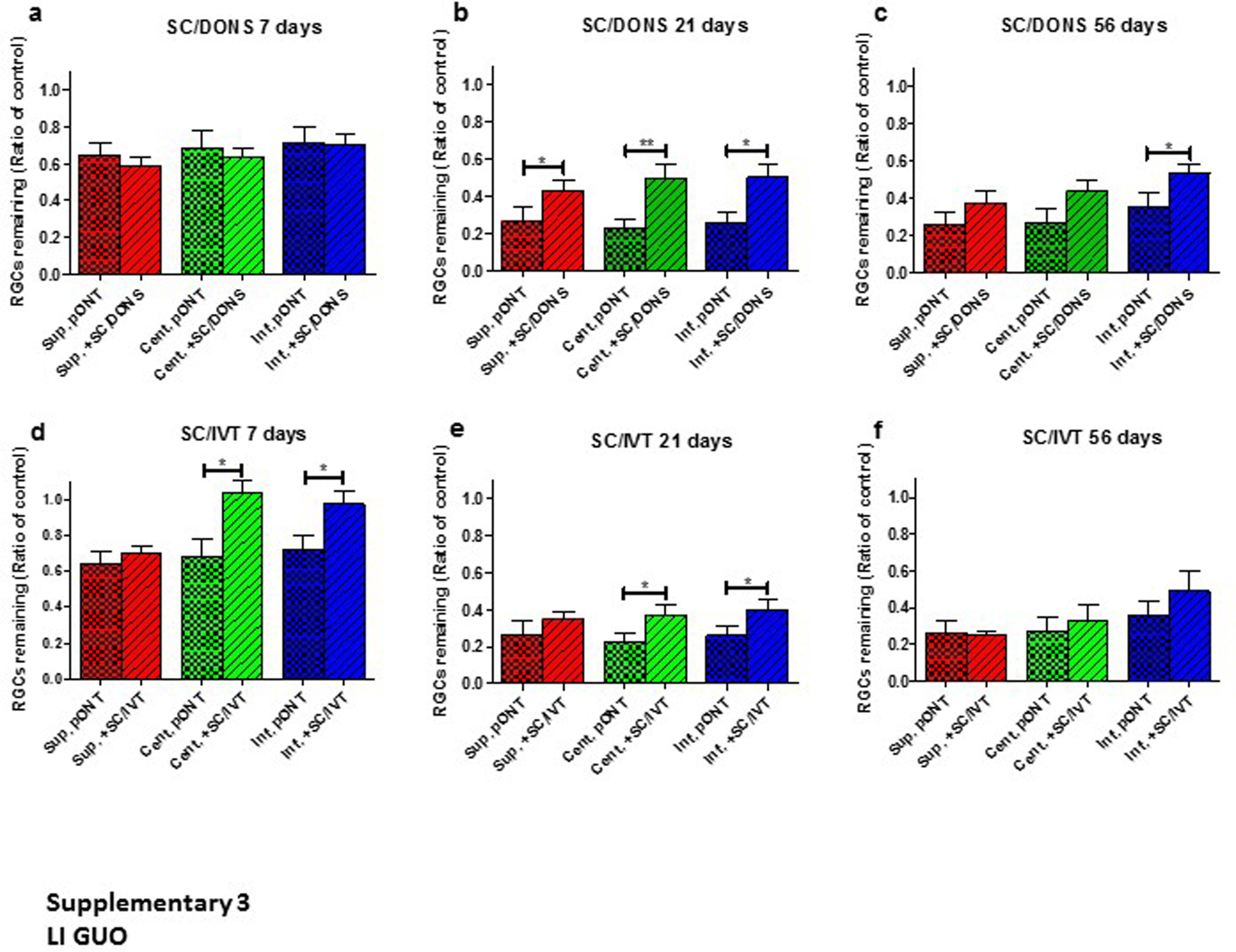

Supplement: Supplementary S3 [file cddis2014399x3.tif]
