# Supplementary material for: Direct optic nerve sheath (DONS) application of Schwann cells prolongs retinal ganglion cell survival in vivo
Source: Cell Death Dis. 2014 Oct 16;5(10):e1460–. doi: 10.1038/cddis.2014.399 (PMC4237238; doi:10.1038/cddis.2014.399)
Supplement: Supplementary Legend [file cddis2014399x4.doc]

**Supplementary Legends**

**Supplementary 1 Comparison of the algorithm with manual RGC counts.** The number of RGCs in retinal whole-mounts was analysed using our newly established algorithm in ImageJ. To validate the algorithm, RGCs in 0.92 mm2 retinal sections were manually counted by 4 trained individuals (manual count). (a) Correlation of algorithm output to average manual counting fitted a straight line. (b) Bland-Altman plot shows correlation between algorithm and manual counts before the correction factor was applied. (c) The table shows the comparison of the algorithm count with manual count (4 masked observers). Pearson’s correlation coefficient for the algorithm count versus the mean manual count was 0.9860, p<0.0001 and the R2 was 0.9722, indicating a highly significant correlation between the techniques. Application of the correction factor to the algorithm results (see Fig. 3e, 3f) eliminated any bias (from -85.68 to 0.4197) and substantially reduced the range (from 405.8 to 310.1), suggesting that this algorithm is better than manual counting in addition to greater time efficiency.

**Supplementary2 No significant effect was seen with vehicle controls on RGC survival.** (a) Vehicle (Matrigel) DONS application (V/DONS) showed no significant effect on RGC density at 21 days compared to pONT only. In comparison, SC/DONS

significantly increased the number of surviving RGCs. (b) Vehicle (culture medium) intravitreal injection (V/IVT) had no significant effect on RGC survival at 7 days compared to pONT only, whilst SC/IVT significantly promoted RGC survival. Data was presented as means ± standard error (SE). **P*<0.05; ***P*<0.01

**Supplementary 3 Comparison of SC/DONS with SC/IVT transplantation on RGC survival in segmented retina.** (a-f) SC/DONS and SC/IVT treatments at 7 (a, d), 21 (b, e) and 56 (c, f) days, respectively, following pONT. SC/DONS showed no effect at 7 days (a), but significantly increased the ratio of RGC survival in all three regions at 21 days (b) and the inferior retina at 56 days (c). In comparison, SC/IVT resulted in a significant effect earlier at 7 days in the central and inferior retina (d). This persisted until 21 days (e) but was no longer present at 56 days (f). Data was presented as means ± standard error (SE). **P*<0.05, ***P*<0.01
